# Imaging by Magnifying Endoscopy with NBI Implicates the Remnant Capillary Network As an Indication for Endoscopic Resection in Early Colon Cancer

**DOI:** 10.1155/2011/242608

**Published:** 2011-02-10

**Authors:** Shoichi Saito, Hisao Tajiri, Tomohiko Ohya, Toshiki Nikami, Hiroyuki Aihara, Masahiro Ikegami

**Affiliations:** ^1^Department of Endoscopy, The Jikei University School of Medicine, 3-25-8, Nishi-Shinbashi, Minato-Ward, Tokyo 105-8461, Japan; ^2^Division of Gastroenterology and Hepatology, Department of Internal Medicine, The Jikei University School of Medicine, Tokyo 105-8461, Japan; ^3^Department of Pathology, The Jikei University School of Medicine, Tokyo 105-8461, Japan

## Abstract

*Introduction*. This study examined whether magnifying endoscopy with NBI observation (ME-NBI) could be useful selecting the appropriate treatment for submucosal invasive cancer (SM cancer). *Patients and Methods*. We analyzed 515 cases of colon tumors excised endoscopically or surgically. We classified capillary network pattern into four types according to the degree of dilatation, irregularity, and distribution of microcapillary features. *Results*. The comparison of capillary pattern and histological features revealed microcapillary networks by using confocal laser-scanning microscopy and ME-NBI in intramucosal lesion or SM cancer with remnant neoplastic glands at the superficial layer. In contrast, the network was absent in SM cancer with desmoplastic reactions, which invaded deeper into the submucosal layer. *Conclusions*. The remaining microcapillary network is designed to maintain the architecture of neoplastic glands. Consequently, loss of this network could correlate with depth of tumor invasion and desmoplastic reaction. Therefore, we can decide the appropriate treatment by using ME-NBI method.

## 1. Introduction

Recent technological advances in endoscopy have supported a flood of new diagnostic methods, collectively reported as “Consensus Terminology” [1]. The narrow band imaging (NBI) used in this study fits into the well-studied image-enhancing endoscopy (IEE) category [2–25] as autofluorescence imaging (AFI) [21–24], as distinct from standard imaging with white light.

There is almost uniform agreement that magnifying endoscopy combined with crystal violet (CV) staining is useful for pit pattern delineation [26–42]. However, this diagnostic imaging technique is not widely used because it is troublesome and time-consuming, and because mucus on the lesion surface may obstruct image acquisition. Magnifying endoscopy with NBI observation (ME-NBI), by contrast, does not involve a complicated technique and provides vascular information in a simple way. ME-NBI provides a simple and fast alternative for vascular imaging that is widely used to examine tumors in the upper gastric tract including the esophagus and stomach for diagnostic purposes [2–7]. NBI has also been used successfully for endoscopic examination of the lower gastrointestinal tract [8–25]. In Japan; however, the capillary network classification using ME-NBI is not popular due to its complication and each medical institution tends to adopt their own classification [11–14]. An important point with NBI observation is establishing early in the diagnosis whether the cancer is best resected endoscopically or surgically. If this point can be resolved, ME-NBI has great potential as an additional tool in the diagnosis and treatment of colon cancer.

This study histopathologically evaluated submucosal invasive cancer (SM cancer) by ME-NBI examination in comparison to the images obtained by confocal laser-scanning microscopy, as a simple and useful means of selecting the appropriate therapeutic intervention.

## 2. Patients and Methods

The study analyzed 515 colon tumors in patients who underwent endoscopic or surgical resection at The Jikei University Hospital from September 2005 to March 2010. These lesions were preoperatively performed with examination by using ME-NBI and magnifying observation CV staining by two experienced specialists (S.S., T.N.) with more than 15 years of endoscopy experience. CF-H260AZI and CF-FH260AZI endoscopes were used in this study (Olympus Medical Systems Co., Ltd Tokyo, Japan).

**Figure 1:**
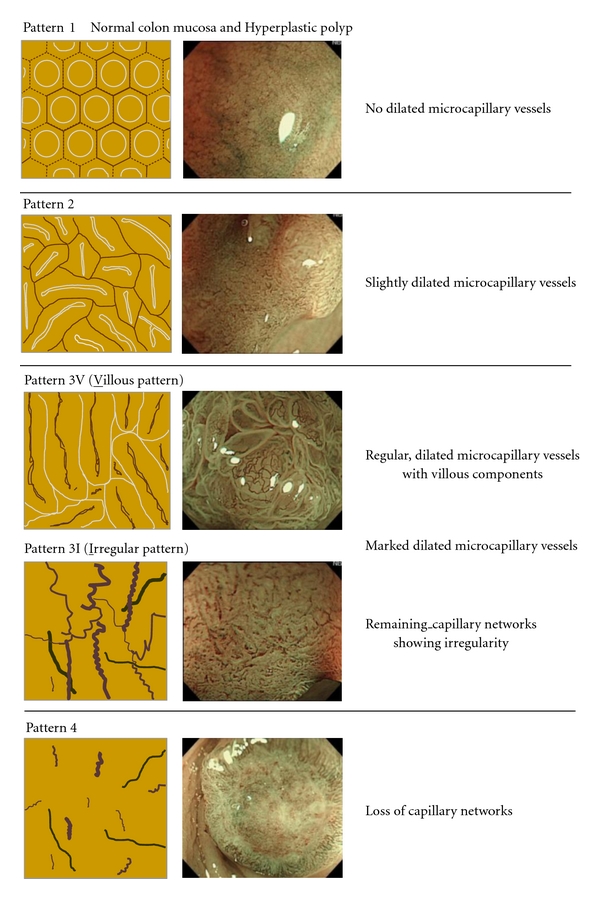
Classification of capillary patterns. Pattern 1: No dilated microcapillary vessels in mucosa. Pattern 2: Regular, smooth microcapillary vessels, slightly dilated. Pattern 3V: Regular dilated capillary vessels in the stromal area with villous component. Pattern 3I: Capillary network preserved by markedly dilated vessels that resemble corkscrew. Pattern 4: No capillary network observed.

Figure 1 depicts the capillary-pattern classification into four groups used in this study; pattern 1 capillaries followed an unrecognized course; pattern 2 capillaries were slightly dilated; pattern 3 capillaries were markedly dilated; pattern 4 was characterized by sparse capillaries not following an obvious vascular course in the lesion. Pattern 3 was further divided into 2 subtypes, whereby one subtype of capillaries assumed a regular course, while the other featured irregularities such as capillary tortuousness, abrupt caliber change, and heterogeneity in shape [7]. Since the type IV pit pattern is observed indirectly in the former subtype, along with a villous component, capillaries of this type were classified as a 3-villous pattern (pattern 3V). The latter subtype was designated as a 3-irregular pattern (pattern 3I).

SM cancer was classified as slight (SM-S) if the depth of invasion was ≤1000 *μ*m or massive (SM-M) cancer if the depth of invasion exceeded 1000 *μ*m according to the General Rules for Clinical and Pathological Studies on Cancer of the Colon, Rectum and Anus [43]. Cancers were measured as recommended by the Japanese Society for Cancer of the Colon and Rectum.

The resected specimens were fixed in 10% buffered formalin for 24 hours. The specimens were cut into 2 mm to 3 mm blocks. Pathological examinations were performed on HE stain by a single pathologist (M.I.), with extensive experience in gastrointestinal tract histopathology without prior knowledge of the endoscopic findings. 

For immunofluorescent microscopy, cells were fixed with 1% paraformaldehyde in a 0.1 M phosphate buffer for 30 min each at 4°C. The specimens fixed in this way were washed in phosphate-buffers saline (PBS) and treated with 0.5% Triton X-100 in PBS for 5 min at 4°C. Indirect immunofluorescent microscopy was then performed. after incubation for 30 min at room temperature with a blocking solution containing 1% normal goat serum in PBS, the specimens were incubated in a blocking solution containing mouse monoclonal antihuman endothelium antibody (CD34; Novocastra Laboratories, Ltd. Newcastle, United Kingdom) for 60 min at room temperature, and then placed at 4°C after extensive washing in PBS, the cells were labeled with fluorescein isothiocyanate-(FITC-) conjugated goat anti-mouse IgG (Molecular Probes, Inc., Eugene, Oregon, USA) in the blocking solution for 60 min at room temperature. After washing in PBS, the specimens were embedded in 50% glycerol in PBS. The specimens were examined using confocal laser-scanning microscopy (LSM 510; Carl Zeiss Co, Jena, Germany). Excitation fluorescein was achieved with an argon laser at a wavelength of 488 nm. An image analysis processing software LSM 5 Image Browser (Carl Zeiss) was used for 3D reconstruction of the immunostained microvessel.

The proportions of histological findings and constructions of lesions within the capillary pattern groups were compared by using the *x* ^2^ test as statistical analysis. To determine differences in the mean distance of invasion into SM layer, comparisons between the capillary pattern groups were performed by one-way analysis of variance (ANOVA), followed by multiple comparison testing using the Bonferroni-Dunn method. Statistical significance was defined as *P* < .05.

## 3. Results

### 3.1. Capillary Patterns As Determined by Laser Scanning Microscopy (LSM) (Figures 2–4)

**Figure 2:**
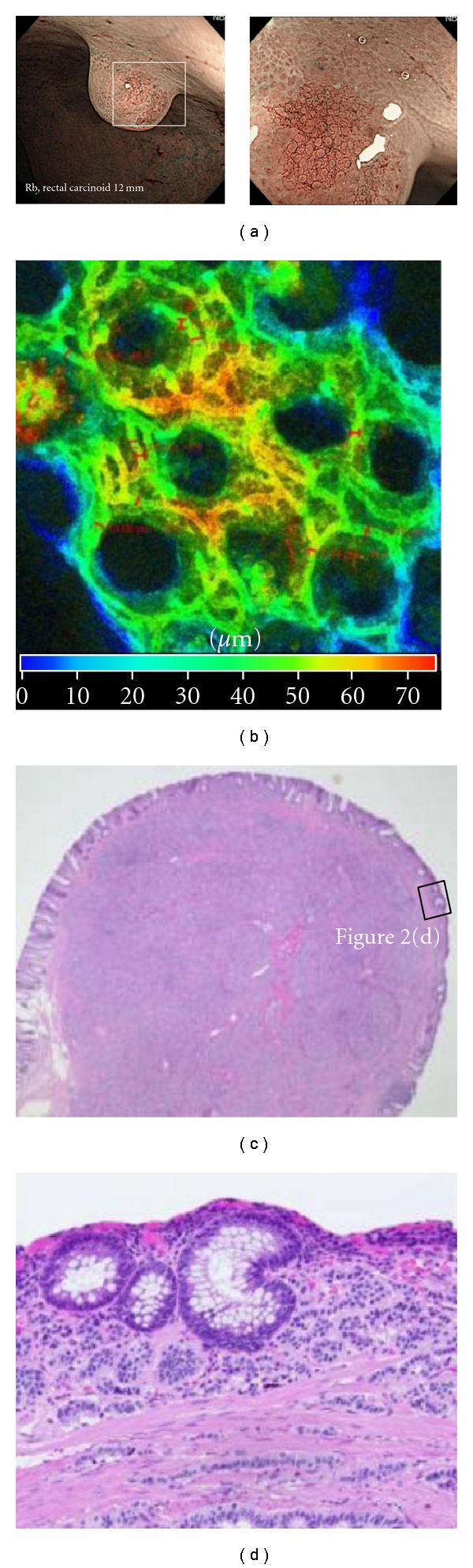
Normal colon mucosa containing carcinoid tumor. (a) The microcapillary vessels are not usually observed using NBI systems in normal colon mucosa. This magnifying image shows microcapillary network in normal colon mucosa (NCM) removed surface epithelium (white box). (b) Three-dimensional structure of the microcapillary network surrounding normal glands displayed using laser scanning microscopy (LSM) in NCM. This finding corresponded with the image obtained by NBI systems. (c, d) Histological view of exposed microcapillary vessels at the surface of mucosa containing carcinoid tumor cells without absorptive tissues (black box).

**Figure 3:**
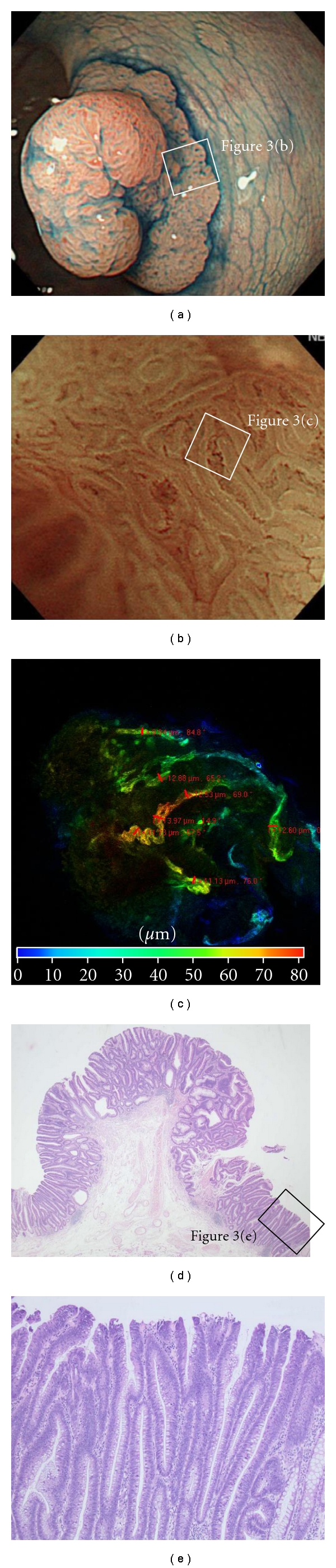
Neoplastic lesion. (a) Endoscopic image of protruding-type colon polyp. (b) Magnifying endoscopy with NBI observation (ME-NBI) showing slightly dilated microcapillary vessels at the tumor edge. (c) LSM imaging showed the corkscrew-like dilated and meandering vessels. (d, e) Histological view revealed tubular adenoma with high-grade dysplastic change by HE stain ((c) low power view, (d) high power view).

**Figure 4:**
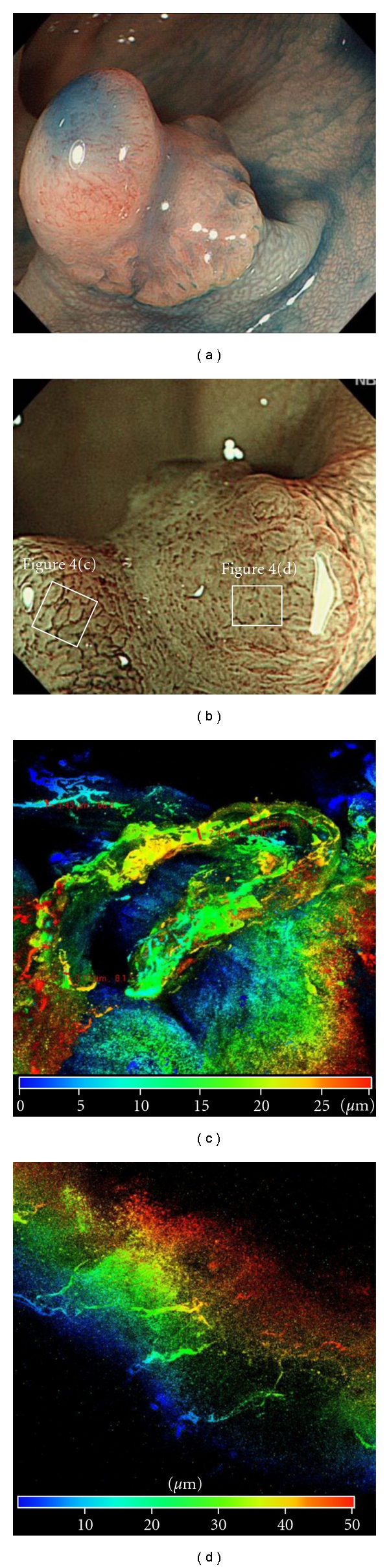
SM cancer with persistent intramucosal lesion. (a) Endoscopic image of protruding-type tumor with a depressed lesion. (b) representative ME-NBI showing marked dilated microcapillary at the protrusion edges on the left side. These congestive vessels form an oval around the gland pits. In contrast, no capillary vessel network remained at depressed lesion. (c) A representative LSM image showing the minimum narrow vessels gathered into a bundle around the gland pits. (d) In the depressed lesion, it was revealed small capillary vessels shaped abrupt caliber change has not left any longer by using LSM.

Capillaries in the normal colon mucosa, tubular adenoma, intramucosal cancer, and SM cancer were examined for course and caliber. A honeycomb-like network was formed by minute vessels of approximately 10 *μ*m in diameter in the normal colon mucosa (Figures 2(a) and 2(b)). Vascular dilation to about 12.11 *μ*m in diameter was observed by LSM (Figure 3(c)) in tubular adenomas. In the tubular adenoma, vascular dilation was brought into view by ME-NBI (Figure 3(b)), and at the sites of notable dilation (Figure 4(b)) thin vessels were found arranged in bundle form on an enlarged view, and vessels were as thin as 3 to 4 *μ*m individually but formed bundles which observed like dilated vessels 40 to 50 *μ*m in diameter (Figure 4(c)). At sites where no apparent capillary course was seen by ME-NBI, only a few minute vessels could be observed travelling from the superficial layer of the lesions to deeper into the submucosa (Figure 4(d)), but their caliber could not be measured.

### 3.2. Relationship between Capillary Pattern and Histological Type or Depth of Invasion (Figures 1 and 5)

In the capillary pattern 1 group, large hyperplastic polyps (HP; 15 mm or larger) accounted for 82.4% of lesions, with serrated adenomas (SA) 14.9%, and tubular adenomas (TA) 2.7%. In the group of capillary pattern 2 lesions showing slight dilation, HP and SA accounted for only about less than 3.0%, with 60.0% of the patterns observed in TA, and remaining 5 lesions (2.8%) being SM cancers. In the group of 3V capillary pattern lesions (villous component showing notable dilation), SA accounted for 4.3% of lesions, TA 19.4%, and intramucosal cancers for the remaining 62.6%. The frequency of atypical lesions was higher in the pattern 3V than pattern 2 lesions group. Furthermore, 13.7% of study lesions were identified as SM cancer and 7.9% as SM-M cancer. In the group of subtype 3I lesions (irregular vascular course), on the other hand, intramucosal lesions decreased to 18.2%, with SM-S and SM-M cancers representing 22.7% and 56.1%, respectively. In the group of pattern 3I lesions, about 50% were indicated for surgical resection. In the type 4 lesions (untraceable capillary course), intramucosal lesion accounted for 1.6% of the total number and this case was diagnosed as moderately differentiated adenocarcinoma. The remaining 98.4% of lesions were SM cancer, with 96.8% of these being, SM-M cancer.

### 3.3. Relationship between Superficial Histological Characteristics and Capillary Patterns (Figures 6 and 7)

The characteristics of histological invasive findings in SM cancers (*n* = 133) were classified into three types: type A showed an intramucosal lesion across the entire area of tumor; type B showed a ruptured muscular mucosa permitting the invading SM end to be exposed to the superficial layer, but no accompanying desmoplastic reaction; type C showed a desmoplastic reaction and were thus analyzed with respect capillary pattern.

All lesions in the group with capillary pattern 2 lesions were of type A, while only 47.1% of lesions in the pattern 3I group were type A, with type B accounting for 47.1% of lesions, and type C 5.8%. In the capillary pattern 4 group, type B accounted for 24.1% of lesions with the reminder (75.9%) classified as type C.

### 3.4. Rate of Accurate Diagnosis of Early Colon Cancer as Determined by Pit Pattern and Capillary Pattern (Table 1)

The rate of accurate diagnosis was determined prospectively based on the findings of vascular dilation between the pit and the capillary patterns in 291 lesions of early colon cancer at the preoperative examination. When the highly irregular type V irregular (V_I_) pit pattern or type V nonstructure (V_N_) pit pattern was selected as pit pattern-related indicators of SM-M, sensitivity and specificity were 94.5% and 86.2%, respectively, with no appreciable difference between V_I_ and V_N_ pits. In contrast, sensitivity and specificity were 95.6% and 77.3%, respectively, when patterns 3I and 4 were selected as the indicators of SM-M. Accuracy rate as an indicator was not significantly different from pit pattern (91.4%) and capillary pattern (88.7%).

## 4. Discussion

An appropriate therapeutic modality for the resection of colon cancer is selected according to criteria established by the Japanese Society for Cancer of the Colon and Rectum in Japan. These criteria consider cancer of the colon and rectum an indication for endoscopic resection if the depth of invasion into the SM layer is ≤1000 *μ*m and no vessels are involved [42–45].

Preoperative diagnosis is of overriding importance from both a clinical and economic standpoint as it may avoid unnecessary treatment. As part of this, a thorough endoscopic examination is considered necessary for selecting the appropriate therapeutic modality. Examination by magnifying endoscopy with CV staining is one such modality, although time limitations can preclude this technique in some cases. Endoscopic equipment has improved markedly with respect to resolution in recent years such that pit pattern can be clearly visualized. As a result, magnifying observation is rarely needed to distinguish SM cancer from HP or TA.

ME-NBI differs from magnifying observation with CV staining in that images are easily interchangeable with the former technique. ME-NBI is also extremely useful for examining capillaries for dilation to differentiate, which is particularly valuable for differentiating a tumor from a nontumorous lesion. To this end, Sano et al. [8, 9, 11] proposed a classification into 3 types for capillary pattern observed by ME-NBI, based on the initial degree of microcapillary dilatation at the mucosal surface. In contrast, another study proposed that assessing the degree of the dilatation of the capillary vessel dilatation should include the pit pattern, which is indirectly observed by ME-NBI, to distinguish between SM-S and SM-M cancer [12]. Based on the present study, we alternatively propose that classifications of capillary pattern, including ours, are difficult to use without experience, because they are complicated patterns and the estimation is quite subjective of endoscopists. Hence a simpler means of endoscopic examination by ME-NBI is needed to enable meaningful selection of the appropriate treatment. 

 In the present case, nearly 90% of patter 1 lesions without vascular dilation were hyperplastic polyps. The tumorous lesions included traditional serrated adenomas [46] and also seemed to include sessile serrated polyps/adenomas [47, 48]. Given that the concept of sessile serrated polyps/adenoma remains to be fully accepted by Japanese pathologists, further investigation and consideration are warranted.

The distance of the capillary pattern observed by ME-NBI from the mucosal surface remains unclear. Thus, we used LSM to accurately resecte the specimen by endoscopic forceps [2, 5], and the LSM images were comparable to those by ME-NBI. Therefore, it seemed that the observation by ME-NBI does represent the surface histological architecture of the mucosa. 

Lesions were classified as pattern 3 or 4 according to the vascular-course details. This is an important point of difference that will help beginners to interpret findings obtained by ME-NBI. For that reason, we classified lesions without a recognizable vascular course into a separate category called pattern 4. LSM imaging confirmed that these lesions were virtually without microcapillary vessels, further indicating that the desmoplastic reaction occurs on the surface of the tumor due to the invasion into the SM layer. 

Lesions of pattern 3 were also divided into patterns 3V and 3I, with the former predominately intramucosal and thus clearly indicated for endoscopic resection. On the other hand, 50% or less pattern 3I lesions were also indicated for endoscopic resection even if they had an irregular vascular course and intramucosal or SM invasion depth within 1000 *μ*m. These lesions showed limited desmoplastic reaction and were left with neoplastic glands leading to the surface layer of the tumor.

In lesions of pattern 4, the vascular course is already devastated and surgical resection should be selected without hesitation when such findings are obtained. The selection of an appropriate therapeutic modality would therefore rest with the presence or absence of a vascular course.

In the case of pattern 3I lesions, however, more than 50% were from cases of SM-M cancer. In such cases where selecting an appropriate modality remains difficult, magnifying observation of the pit pattern with conventional CV staining and endoscopic ultrasound examination should be combined with due discretion.

As discussed above, microcapillary patterns do not directly reflect the depth of cancer invasion, and ME-NBI observation can accordingly be considered a useful aid to pit pattern diagnosis. Endoscopic resection may be selected as a temporary measure for the treatment of pattern 3I lesions that are suspected of invasion by SM-M cancer if patients have a tumor in the lower part of the rectum or serious complications in the cardiovascular or respiratory systems.

## Figures and Tables

**Figure 5:**
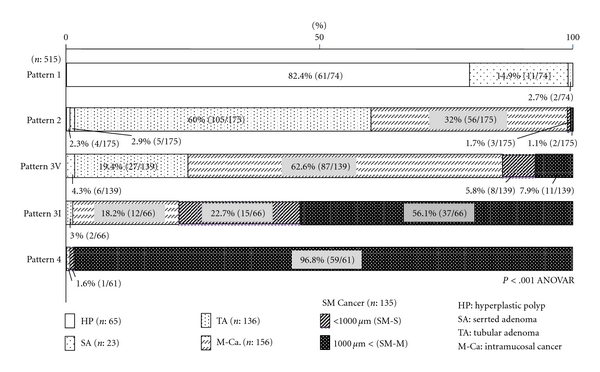
Comparison between capillary pattern and histological findings.

**Figure 6:**
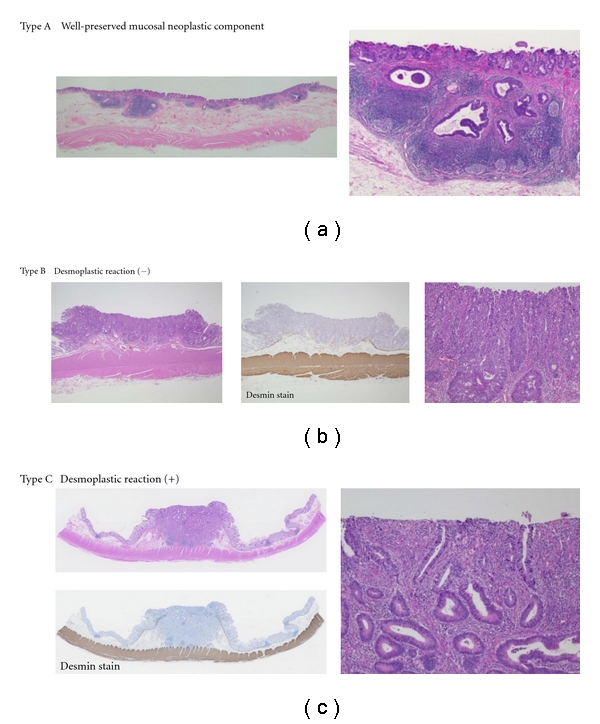
Classification of SM cancer according to superficial histological features.

**Figure 7:**
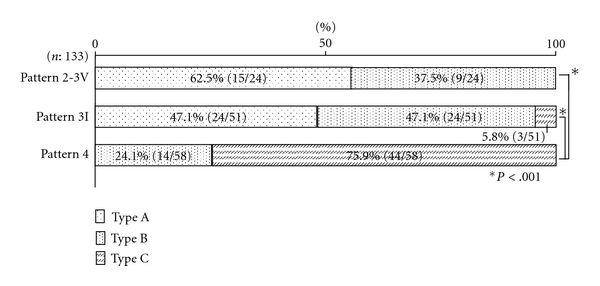
Comparison between capillary pattern and superficial histological characteristics in SM cancer.

**Table 1:** Comparison of diagnostic accuracy with capillary pattern and pit pattern on intramucosal cancer and SM cancer.

	Sensitivity	Specificity	Accuracy
Capillary pattern	95.6%	77.3%	88.7%
Pit pattern	94.5%	86.2%	91.4%

			(*n*: 291)

## Abbreviations

AFI: Auto fluorescence imaging;
IEE: Image enhancing endoscopy;
NBI: Narrow band imaging;
ME-NBI: Magnifying endoscopy with NBI observation;
NCM: Normal colon mucosa;
SM cancer: Submucosal invasive cancer;
CV: Crystal violet;
LSM: Laser scanning microscopy.
